# Carbon Dioxide Mediates the Response to Temperature and Water Activity Levels in *Aspergillus flavus* during Infection of Maize Kernels

**DOI:** 10.3390/toxins10010005

**Published:** 2017-12-22

**Authors:** Matthew K. Gilbert, Angel Medina, Brian M. Mack, Matthew D. Lebar, Alicia Rodríguez, Deepak Bhatnagar, Naresh Magan, Gregory Obrian, Gary Payne

**Affiliations:** 1 USDA/Agricultural Research Service, 1100 Robert E Lee Blvd., New Orleans, LA 70124, USA; brian.mack@ars.usda.gov (B.M.M.); matthew.lebar@ars.usda.gov (M.D.L.); deepak.bhatnagar@ars.usda.gov (D.B.); 2 Applied Mycology Group, Cranfield Soil and Agrifood Institute, Cranfield University, Bedfordshire K43 0AL, UK; a.medinavaya@cranfield.ac.uk (A.M.); n.magan@cranfield.ac.uk (N.M.); 3 Food Hygiene and Safety, Meat and Meat products Research Institute, University of Extremadura, 10003 Caceres, Spain; aliciarj@unex.es; 4 Department of Entomology and Plant Pathology, 223 Partners III, P.O. Box 7567, North Carolina State University, Raleigh, NC 27695, USA; grobrian@ncsu.edu (G.O.); gaplab@ncsu.edu (G.P.)

**Keywords:** aflatoxin, *Zea mays*, climate change, secondary metabolites, RNA-seq

## Abstract

*Aspergillus flavus* is a saprophytic fungus that may colonize several important crops, including cotton, maize, peanuts and tree nuts. Concomitant with *A. flavus* colonization is its potential to secrete mycotoxins, of which the most prominent is aflatoxin. Temperature, water activity (a_w_) and carbon dioxide (CO_2_) are three environmental factors shown to influence the fungus-plant interaction, which are predicted to undergo significant changes in the next century. In this study, we used RNA sequencing to better understand the transcriptomic response of the fungus to a_w_, temperature, and elevated CO_2_ levels. We demonstrate that aflatoxin (AFB_1_) production on maize grain was altered by water availability, temperature and CO_2_. RNA-Sequencing data indicated that several genes, and in particular those involved in the biosynthesis of secondary metabolites, exhibit different responses to water availability or temperature stress depending on the atmospheric CO_2_ content. Other gene categories affected by CO_2_ levels alone (350 ppm vs. 1000 ppm at 30 °C/0.99 a_w_), included amino acid metabolism and folate biosynthesis. Finally, we identified two gene networks significantly influenced by changes in CO_2_ levels that contain several genes related to cellular replication and transcription. These results demonstrate that changes in atmospheric CO_2_ under climate change scenarios greatly influences the response of *A. flavus* to water and temperature when colonizing maize grain.

## 1. Introduction

*Aspergillus flavus* is a saprophytic fungus that infects several crops of agronomic importance, including corn, cotton, peanuts and tree nuts. Prior to harvest, *A. flavus* may infect the fruiting bodies or seeds in crops and produce several toxic secondary metabolites, including the polyketide derived aflatoxins (AFs), cyclopiazonic acid and aflatrem. In the USA and other industrialized countries, the establishment of contamination thresholds by regulatory agencies and close monitoring of crops have minimized the direct impact on human health. However, economic losses remain significant. Regarding AF contamination in the USA alone, estimates of losses are between $163 million for maize crops to $500 million annually for maize, peanuts and other crops [1,2]. In lower middle income countries (LMCs) where the regulatory controls either do not exist or are not enforced, especially in sub-Saharan Africa, the consumption of food contaminated with AFs are directly linked to liver disease, tumor development, stunted development in children and other medical defects (reviewed in [3]). The AF compounds produced by *A. flavus* include the structurally similar forms B_1_, B_2_, G_1_ and G_2_. Most *A. flavus* strains produce only the B forms of AF, however other related species, such as *A. parasiticus* and *A. nomius*, produce both B and G AFs [4,5]. 

Mitigation strategies to minimize contamination of maize have involved the implementation of different approaches. These include the development of genetically modified (GM) maize crops [6,7], which are resistant to pests and minimize damage that provides entry points for *A. flavus*; classic breeding for resistance [8], and pre-harvest biocontrol by using naturally non-aflatoxigenic strains of *A. flavus* to outcompete the toxigenic strains [9]. The latter approach has resulted in several commercial biocontrol products currently being used in maize, groundnuts and cotton. 

Environmental factors, including water activity (a_w_), temperature, light, as well as their interactions, have been demonstrated to have a significant influence on germination, growth and AFs production by strains of *A. flavus* [10,11,12,13]. Recent studies by Medina et al. (2015) demonstrated that water activity had a statistically significant effect on growth rates, whereas the effect of temperature and CO_2_ was negligible under the conditions tested. Further studies by Medina et al. (2017) examined the impact of a_w_ and temperature interactions on global transcriptomic changes in *A. flavus* and how this relates to AFB_1_ production in maize-based matrices. 

There has been interest in the resilience of mycotoxigenic fungi in relation to potential future climate change (CC) scenarios and whether interactions between a_w_,increased temperature andelevated CO_2_ might impact mycotoxin production at a fundamental genomic level and phenotypic toxin production. By the year 2100 temperatures could increase by 4 °C, and CO_2_ levels are anticipated to reach approximately 1000 ppm, depending on mitigation efforts [14]. Medina et al. (2015) showed that when *A. flavus* was exposed in vitro to these three-way interacting CC factors growth was unaffected, while expression of biosynthetic genes (*aflD*; *aflR*) and phenotypic AFB_1_ production were significantly increased. This is likely to result in economic impacts. For example, it has been suggested that CC could increase economic losses in the maize industry by $50 million to up to $1.7 billion if AF production increases under such scenarios [15]. A recent review suggested that food security, especially in LMCs, could be profoundly impacted on due to such increases in mycotoxin contamination of staple food commodities [16]. 

There has been interest in applying current technologies, including functional genomics, transcriptomics, and proteomics to measure the impact of interacting CC-related abiotic parameters on fungal growth, the regulation of the AF biosynthetic cluster, and toxin production. A recent study examined the impact of interactions between a_w_ and temperature in the transcriptome of *A. flavus* when colonizing maize [17]. It was determined that a_w_ (0.99, 0.91) and temperature (30, 37 °C) influenced the colonization of maize grain by *A. flavus* with a significant effect on AFB_1_ production at 0.91 a_w_ and 37 °C. Both environmental factors affected biological processes and the numbers of up- and downregulated genes. The interacting environmental factors influenced the functioning of the secondary metabolite clusters for AFs and CPA such that an elevated number of genes were co-regulated by both a_w_ and temperature. For example, an interaction effect for 4 of the 25 AFB_1_ genes, including regulatory and transcription activators occurred. For CPA, all 5 biosynthetic genes were affected by a_w_ stress, regardless of temperature. 

The objectives of the present study were to examine, for the first time, the impact of interactions between a_w_ (0.99, 0.91), temperature (30, 37 °C) and CO_2_ (350, 650 and 1000 ppm) on *A. flavus* (NRRL 3357) colonization of stored maize grain to evaluate the impacts on (1) the whole genome, including the aflatoxin biosynthesis gene cluster using RNA-seq, (2) effects on key biosynthetic genes using q-PCR, and (3) effects on AFB_1_ production. We used functional genomics to better understand the transcriptomic response of the fungus to CC parameters of a_w_, temperature and CO_2_ levels, with a view towards predicting changes in fungal infection and toxin production associated with resilience to such climatic stress factors. 

## 2. Results

### 2.1. The Interaction of Water, Temperature, and CO_2_ Impact AFB_1_ Production in Maize Grain

**Figure 1:**
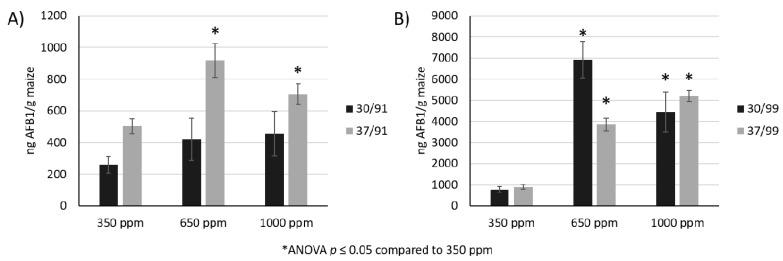
Aflatoxin B_1_ (AFB_1_) production by *A. flavus* under different combinations of environmental conditions. (**A**) At 30 °C and under low water activity levels of 0.91 a_w_ (“30/91”), the effects of CO_2_ are minimal; however, at 37 °C (“37/91”) there are increases in AFB_1_ production correlating with higher CO_2_ levels. (**B**) At high water activity levels the biosynthesis of AFB_1_ was significantly elevated at both 30 °C (“30/99”) and 37 °C (“37/99”). ANOVA: analysis of variance statistical analysis. * *p* ≤ 0.05.

Overall, AFB_1_ production showed a positive correlation with water availability and CO_2_ levels. AFB_1_ was extracted and quantified after 10 days incubation at 30 °C or 37 °C, 0.91 or 0.99 a_w_, and 350, 650, or 1000 ppm CO_2_. At low a_w_ (Figure 1A) the quantity of AF produced was 5- to 10-fold lower than at high a_w_ levels (Figure 1B). However, the effect of CO_2_ was positively correlated with AFB_1_ production at both high and low a_w_ levels. With an elevated CO_2_ level of 650 ppm, there was an interaction effect with temperature and a_w_. At 0.91 a_w_, AFB_1_ production was highest at 37 °C. Conversely, at 0.99 a_w_ the temperature condition of 30 °C exhibited higher toxin levels. The trends observed indicate that CO_2_ affects toxin production. The maximum quantity of toxin was observed at elevated levels of CO_2_ (650 ppm) with low water activity and at 30 °C, however, no additional accumulation of AFB_1_ was observed at 1000 ppm.

### 2.2. Effect of Three-Way Interacting CC Conditions on Gene Expression

**Figure 2:**
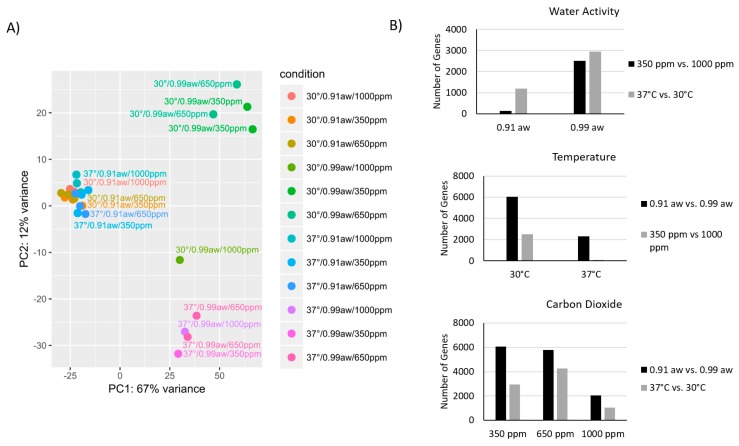
(**A**) Principle component analysis and (**B**) total gene counts of differentially expressed genes indicate that of the three environmental variables tested, water activity is the primary driver of transcriptional changes, with temperature also having a measurable impact. Carbon dioxide levels (1000 ppm vs. 350 ppm) has the largest impact at 0.99 a_w_ (**B**, **top**) and at 30 °C (**B**, **middle**). High carbon dioxide levels (1000 ppm) reduces the level of genes affected by water activity and temperature by approximately 1/3 (**B**, **bottom**).

RNA-seq of *A. flavus* genes showed a large global effect with water and temperature stress, but a limited effect with elevated CO_2_ levels. Between 6.22 × 10^5^ and 3.37 × 10^7^ reads mapped to exogenic regions of *A. flavus* strain 3357 (Table 1). Principle component analysis (PCA) indicated that a_w_ caused the largest variance observed (Figure 2A), accounting for 67% of the variance, however samples with similar water activity cluster together on PC1, indicating there little sample variation for this metric. The second principle component indicated that a change in temperature was the second most important factor, but only for samples stored with freely available water (0.99 a_w_). All samples stored under water stress (0.91 a_w_) clustered together and showed relatively little inter-sample variance. At high a_w_, a change in temperature resulted in a high variance in gene expression. The total number of genes affected under the various conditions are illustrated in Figure 2B. For each data point in Figure 2B, the unidentified variable (a_w_ level, temperature or CO_2_ level) is the baseline condition (30 °C, 0.99 a_w_, or 350 ppm CO_2_) for those samples being compared. Of note, ~10-fold more genes responded to temperature and CO_2_ stress at high a_w_ levels (Figure 2B, upper graph). Likewise, ~3x more genes were affected by temperature and ~25x more genes were affected by CO_2_ in the non-stressed temperature conditions (30 °C; Figure 2B, middle graph). High CO_2_ levels (1000 ppm) had a significant impact on the number of genes expressed, decreasing the number of genes affected by water and temperature by ~3 fold (Figure 2B bottom graph). In summary, elevated temperature, water stress, and increased CO_2_ decreased the number of differentially expressed genes when compared to the non-stressed conditions. 

**Figure 3:**
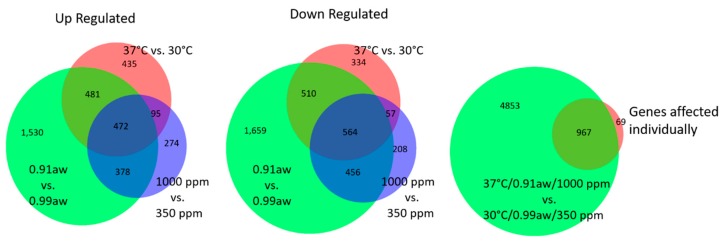
Venn Diagrams illustrate the number of genes up regulated (**left**) and down regulated (**middle**) by individual conditions. For each comparison described, control conditions are assumed (30 °C, 0.99 a_w_ and 350 ppm CO_2_). When all three conditions are changed simultaneously, 5820 genes are affected, however when only one environmental condition is changed, only 967 of these genes are affected, indicating a significant difference between the cumulative and individual effects of environmental changes (**right**).

A significantly larger number of genes were affected by water stress, whereas approximately equal numbers were affected by temperature and CO_2_ (Figure 3, left and center). Most of the genes that were affected by changing temperature and CO_2_ levels were also affected by low a_w_, with 472 genes being upregulated by all three environmental conditions and 564 genes being downregulated. Figure 3 illustrates how changing all three environmental variables simultaneously resulted in a significantly larger number of genes (4853) being affected than changing variables independently. In other words, there is a cumulative effect of changing environmental conditions on global gene expression. The group labeled “Genes affected individually” refers to genes that are affected by just a_w_, temperature, or CO_2_ levels when examined separately. Sixty-nine genes were affected by each of the three conditions individually, but not affected when all three conditions are applied simultaneously.

### 2.3. Effect of Three-Way Interacting CC Conditions on Biological Processes

**Figure 4:**
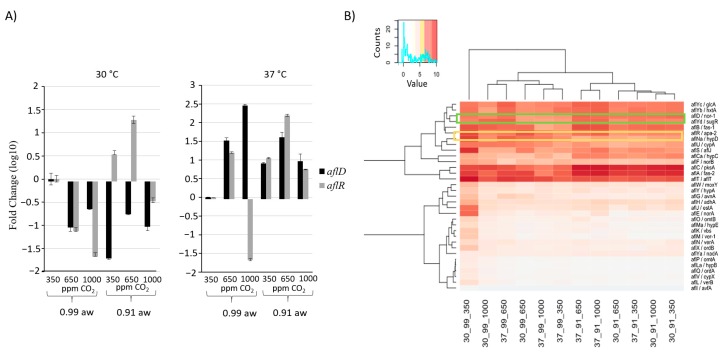
Gene Expression of the aflatoxin gene cluster. (**A**) Quantitative PCR analysis shows the expression of *aflR* and *aflD*, an aflatoxin cluster transcription factor and structural gene, respectively. After 10 days of incubation on maize kernels gene levels at 30 °C generally decrease, however the effects of high CO_2_ (1000 ppm) levels at 0.91 a_w_ indicate decrease values (**left**), possibly in response to elevated AFB_1_ levels. At 37 °C gene levels remain high, however, again at 1000 ppm CO_2_ the transcription factor *aflR* is decreased. (**B**) The heat map of regularized log transformed counts indicate hierarchal clustering associated with water activity levels. The clustering also indicates expression patterns that suggest early genes in the pathway may be responsive to high CO_2_ levels (See Results).

After 10 days *A. flavus* colonization of the maize kernels, quantitative PCR was conducted on two AF cluster genes: *aflR*, the regulating transcription factor, and *aflD*, a reductase (Figure 4A). Gene expression levels are shown relative to a control condition of 30 °C, 0.99 a_w_, and 350 ppm CO_2_. Four important observations were made: (1) at 30 °C/0.99 a_w_ there were decreases in *aflR* and *aflD* expression, even at elevated CO_2_ levels (650 ppm; 1000 ppm) relative to the control (350 ppm). This is in contrast to what was observed at 37 °C, where *aflD* is activated at higher levels in the 650 and 1000 ppm CO_2_ treatments at 0.99 a_w_; (2) in two separate stressed conditions 30 °C/0.91 a_w_ (water stressed conditions) and 37 °C/0.99 a_w_ (high temperature), there are sharp differences in *aflR* expression levels between 650 and 1000 ppm, indicating that some CO_2_ level between the two may represent a threshold; (3) at low a_w_ and high temperature, there were higher levels of activity than at 0.99 a_w_/350 ppm; (4) at 37 °C/0.99 a_w_, a dose response in relation to CO_2_ was observed.

The heat map (Figure 4B) indicates relative expression levels for all genes in the AF gene cluster obtained from RNA-seq. Hierarchical clustering of sample conditions (top brackets) and genes (left side) are indicated. The AF cluster genes *aflD* and *aflR* are clustered relatively close together (genes 3 and 6, respectively), indicating similar expression profiles, which is in agreement with qPCR results where 9 of the 12 samples are similarly expressed. Hierarchical clustering of the samples indicated water availability as the primary determinant of expression patterns. After 10 days, the 30 °C/0.99 a_w_/350 ppm CO_2_ condition exhibited higher overall expression levels, distinct from any of the stressed conditions. Notably, expression at 30 °C/0.99 a_w_/350 ppm is higher than at 650 ppm and 1000 ppm CO_2_. Three of the genes in the AF biosynthesis pathway, fatty acid synthases *aflA* and *aflB*, and the polyketide synthase *aflC*, all of which are responsible for the synthesis of norsolorinic acid, were enriched in all samples. The reductases coded by *aflD* and *aflF*, responsible for the reduction of norsolorinic acid, were also enriched at relatively high levels; however, a third reductase, *aflE*, exhibited the highest enrichment level in the most stressed condition examined (37 °C/0.91 a_w_ and 1000 ppm CO_2_). 

Enrichment analysis was conducted to identify KEGG biological processes with an over-representation of genes being up- or downregulated. Table 2 lists the KEGG categories affected by changes in conditions, both individually and combined, from which several important observations can be made. Primarily, energy-related metabolic processes such as glycolysis/gluconeogenesis, purine/pyrimidine metabolism, and starch/sucrose metabolism are prominent among conditions analyzed. Most genes in this category that are essential for glycolysis, including triosephosphate isomerase (AFLA_094630), fructose bis phosphate aldolase (AFLA_030930), and pyruvate kinase (AFLA_087900) are all downregulated in all the conditions examined. The KEGG categories affected, that are unique to changes in CO_2_ concentration (30 °C/0.99 a_w_/350 ppm CO_2_ vs. 30 °C/0.99 a_w_/1000 ppm CO_2_), are cysteine and methionine metabolism (*p* = 0.009) and folate biosynthesis (*p* = 0.030). Three categories of KEGG were affected only when all three environmental conditions were changed simultaneously (37 °C/0.91 a_w_/1000 ppm CO_2_ vs. 30 °C/0.99 a_w_/350 ppm CO_2_). These were taurine and hypotaurine metabolism (*p* = 0.014), cyanoamino acid metabolism (*p* = 0.017), and ether lipid metabolism (*p* = 0.049).

### 2.4. Effect of CO_2_ and Interactions with Other Abiotic Factors on Secondary Metabolite Gene Clusters

To identify secondary metabolic gene clusters that were affected by the interacting environmental conditions tested according to the RNA-seq results, a Secondary Metabolite Unique Regions Finder (SMURF) analysis of the *A. flavus* genome was conducted. Table 3 lists several SMURF-identified secondary metabolic gene clusters labeled according to Georgianna et al. [18] and their corresponding relative expression values (log_2_ fold change). The full list of secondary metabolite-associated genes identified by SMURF is provided in Supplementary Table S1.

Many of the genes listed in Table 3 have high sequence identity to previously characterized genes (in black). Of note, four of the genes (shown in red) are affected by both of the elevated CO_2_ levels (650 ppm and 1000 ppm). These consist of two dimethylallyl tryptophan synthases (DMTS), a terpene cyclase, and the hybrid NRPS/PKS shown to be responsible for leporin biosynthesis (see Discussion).

### 2.5. Identification of Gene Networks Affected by CO_2_ Levels

**Figure 5:**
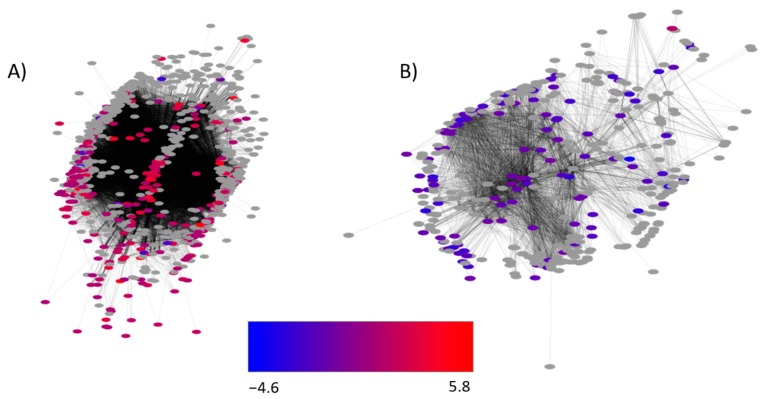
Weighted Gene Network Co-expression Analysis followed by visualization in Cytoscape shows two unidentified networks heavily influenced by increased carbon dioxide levels (1000 ppm CO_2_ vs. 350 ppm CO_2_) at 30 °C and 0.99 a_w_. The color of the node indicates the log_2_ fold change values. (**A**) 268 out of 905 genes in the network are differentially expressed, with most showing increased levels. (**B**) 114 genes out of 415 genes in the network show altered expression levels. See Results for description of the genes in the network.

Gene co-expression networks were determined by analyzing all RNA sequencing results using WGNCA. The results were then visualized using Cytoscape. This analysis revealed two prominent gene networks with significant numbers of genes affected by CO_2_ levels (Figure 5).

Genes with altered differential expression at 30 °C, 0.99 a_w_ and 1000 ppm CO_2_, compared with 350 ppm, are colored and genes not differentially expressed are shown in grey. The network illustrated in Figure 5A shows 905 genes interacting in total, with 268 genes differentially expressed. The network has three distinct clusters on the left, right, and center, and the center cluster is co-expressing with genes in the outer clusters (indicated by black lines (edges)). The left and right clusters (95 and 67 genes, respectively) have edges connecting to the 45 genes in the center cluster. Gene ontology (GO) enrichment analysis of the individual clusters indicated the molecular function of genes on the left involve primarily ATP-binding protein kinases (e.g., GO:0005524, ATP binding; GO:0004672, protein kinase activity) (See Supplemental Table S2). The right cluster is enriched in structural and secretory-related genes (e.g., GO:0000166, nucleotide binding, *Rheb*, and *Myo5*; GO:0051056 regulation of small GTPase mediated signal transduction, *Sar1*). The center of the network contained no enriched GO categories, however it does contain several transcription factors and transcriptional regulatory elements (AFLA_029620, AFLA_114920, AFLA_003630). Figure 5B is a second network (network 2) identified by WGNCA analysis and Cytoscape rendering. It shows 415 genes in the network, 114 of which are significantly affected by changing CO_2_ levels. Network 2 consists of a significantly enriched number of ribosomal-related genes (GO:0003735, structural constituent of ribosome). Other non-ribosomal genes include and Hsp90-binding chaperone *sba1* (AFLA_095590), a mycelial catalase *cat1* (AFLA_090690), and the AF cluster gene *aflT* (AFLA_139420) (Supplementary Table S2).

## 3. Discussion

To date, the majority of research pertaining to environmental effects on AF production, fungal growth, and plant pathogenicity have focused on the effects of water availability and temperature. Work by Medina et al. [19,20] was among the first attempts to examine the effects of a_w_ and temperature in the context of higher CO_2_ levels. They found that the interactive effect of water stress, high temperature and high CO_2_ increased both gene expression of the AF biosynthesis genes *aflR* and *aflD*, and AFB_1_ production in maize kernels. While the experimental procedure here permitted us to observe changes in gene expression and AF production after 10 days of growth, it is limited in that we cannot observe a temporal response of gene activation followed immediately by AF biosynthesis. However, we further expand on previous findings, allowing us to characterize putative secondary metabolic gene clusters, important developmental genes, and identify networks of co-expressed genes affected by CO_2_ levels.

Most evidence involving fungal carbon metabolism involves the release of CO_2_ via cellular respiration, however CO_2_ serves other functions. Hall et al. [21] reported that CO_2_ serves as an intra-colony signaling molecule important for colonization and pathogenesis in the fungus *Candida albicans*. It has also been shown that insects, in symbiosis with fungi, demonstrate preferences for certain ranges of elevated CO_2_ in which to conduct their fungal-rearing [22]. Furthermore, elevated CO_2_ present in the ambient environment of soil samples containing a mixed fungal population decreases respiration activity [23] and decreases AF accumulation in *A. parasiticus* [24]. The data here demonstrates that while marked effects in transcription (and by consequence growth and toxin production) are primarily a result of water availability, and secondarily temperature, changing CO_2_ levels altered fungal response to both water and temperature changes. This is made apparent by the total numbers of genes affected and by our observing that changes brought on by low water or high temperature stresses can vary depending on CO_2_ availability. Furthermore, evidence indicates that the combination of environmental stressors, including high CO_2_, have a compounding effect compared to that of individual stressors. 

The production of secondary metabolites under different environmental conditions is of concern due to potential shifts in toxigenic potential. The biosynthetic gene cluster 19 in *A. flavus* contains a dimethylallyl tryptophan synthase (DMAT), which prenylates tryptophan or other aromatic substrates, however the secondary metabolite biosynthesized has yet to be identified. While the expression profile of individual cluster genes does not necessarily correlate with metabolite biosynthesis, DMATS genes are involved in the biosynthesis of several toxic metabolites produced by fungi that infect and contaminate crops such as cyclopiazonic acid [25] in *A. flavus* and ergotamine in *Claviceps purpurea* [26]. Toxic metabolites can serve offensive or defensive functions, increasing virulence of the fungus toward its host, or protecting the fungus from threats by other microorganisms or predation. The backbone gene in cluster 23, a hybrid Non-ribosomal Peptide Synthase/Polyketide Synthase (NRPS/PKS), is upregulated with increasing CO_2_ levels. This cluster is responsible for the biosynthesis of leporins [27]. Leporin A has anti-insectan properties [28], while leporin B [29] can chelate iron, forming a trimer with Fe^3+^ [27]. Siderophore biosynthesis and siderophore-mediated iron uptake have been found to increase under hypoxic conditions in *A. fumigatus* [30]. AFLA_125760, which was also upregulated at elevated CO_2_ levels, encodes for a terpene cyclase flanked by a putative P450 alkane hydroxylase (AFLA_125750) and a steroid alpha reductase (AFLA_125740). These types of cyclases convert the linear triterpene squalene into cyclized products under hypoxic conditions. Cyclized triterpenes, such as fungal ergosterol and bacterial hopenoids, provide cell membrane structural integrity and fluidity. Production of ergosterol, the most prevalent cyclized triterpene found in fungal cell membranes, is affected by mechanical and oxidative stress [30,31]. Other cyclic triterpenes may also be expressed under hypoxic and stressful conditions to improve cell membrane integrity.

Here we have demonstrated for the first time transcriptome-wide changes that occur in a pathogenic fungus while colonizing maize kernels under these interacting CC-related environmental conditions. AF biosynthesis and genes from the AF biosynthetic cluster responded to elevated CO_2_ levels, as did several other identified secondary-metabolic gene clusters. Further, there is a global change in the transcriptome in response to water and temperature stress under high CO_2_ conditions. This works lays a solid foundation for further research to establish which genes and gene networks should be targeted for fungal inhibition in future CC scenarios. 

## 4. Materials and Methods

### 4.1. Fungal Strain

*Aspergillus flavus* strain NRRL 3357 (ATCC 200026, GenBank assembly accession: GCA_000006275.2) [32,33] was obtained from the Southern Regional Research Center, New Orleans, LA. This strain has been characterized extensively in several areas, including metabolite production [34,35], genome profiling, [36], for the development of biological control [37], molecular ecology [38] and many others. Spore stocks were stored at 4 °C or sub-cultured on Malt Extract agar (MEA; CM59, Oxoid Ltd., Basingstoke, UK) when needed.

### 4.2. Sample Preparation and Treatment

Undamaged French feed maize kernels were used in this study. Initially a standard curve was conducted whereby known amounts of water were added to multiple 10 g samples of kernels, incubated at 4 °C for 48 h, and the resulting a_w_ of the kernels was determined using an Aqualab 4 TE water activity meter (Decagon Devices, Pullman, WA, USA). 

Based on a_w_ standard curve results, a volume of water was added to each 10 g sample of maize kernels to obtain a_w_ levels of 0.99 and 0.91 (minus 200 μL for the later additions of *A. flavus* spore suspension). The maize kernels were then placed in glass culture vessels containing a microporous lid, which allows for moisture and air exchange (Magenta, Sigma Ltd., Castleford, UK). Subsequently, 200 μL of spore suspension (approx. 10^6^ spores/mL) were added to make up the predetermined amounts of water required and thoroughly mixed. The inoculated vessels for each treatment were placed in enclosed environmental chambers. To control humidity levels, 2 glass jars (500 mL) containing glycerol-water solutions, appropriate to maintaining the equilibrium relative humidity at the target a_w_ level, were placed in each chamber. The chambers were incubated at 30 and 37 °C for 10 days. To control CO_2_ and maintain humidity, specialty certified CO_2_ gas cylinders (British Oxygen Company, Jierfude, UK) containing either air, 650 ppm CO_2_ or 1000 ppm CO_2_ were used for flushing the environmental chambers. The gases were bubbled through a glycerol:water solution of the required a_w_ level before flushing through the chambers and the valves closed. CO_2_ flushing was performed every day as described previously [13]. The glycerol-water solutions in the chambers were replaced with fresh solutions every 2 days during the incubation period. Three replicates per treatment were used in all cases. At the end of the incubation period, samples were snap frozen using liquid N_2_ and kept at −80 °C until a portion of each sample could be used for RNA extraction and purification, or dried for AFB_1_ extraction and clean-up prior to quantification using HPLC analysis.

### 4.3. Aflatoxin Analysis

AFB_1_ extraction was performed using AflaStar™^®^—Immunoaffinity Columns (IAC, Romer Labs Inc., St. Louis, MO, USA), following the manufacturer’s instructions. Briefly, 5 g of the sample were dried overnight at 80 °C and stored at room temperature. The samples were ground and 4 g was placed into a 50 mL Falcon tube, to which 16 mL of a methanol:water (60:40 *v*:*v*) solution was added. The samples were shaken for 1 h at room temperature, and then filtered through qualitative filter paper (QL 110, Fisher Scientific UK Ltd., Loughborough, UK). The extract (1 mL) was diluted in a 15 mL Falcon tube with 9 mL of PBS buffer (0.05 M/0.15 M NaCl, pH 7.4, Fisher Bioreagents^®^, Fisher Scientific UK Ltd., Loughborough, UK), and pH was checked with pH strips. The diluted extract was applied to the IAC, and allowed to drip through. After further cleaning, 3 mL of Methanol (HPLC grade) was used to elute the AFs. The eluent was dried and standards were prepared using 200 μL AF (R-Biopharm Rhône Ltd., Darmstadt, Germany) stock solution comprised of 1 ng/uL AFB_1_. The stock solution was pipetted into 2 mL Eppendorf tubes and left to evaporate to dryness overnight inside a fume hood. For quantification of AFs, 200 μL hexane was added to the residue followed by the addition of 50 μL triflouroacetic acid (TFA). The mixture was then vortexed for 30 s and then left for 5 min. Thereafter, a mixture of water:acetonitrile (9:1, *v*:*v*) was added and the entire contents of the tube were vortexed for 30 s, after which the mixture was left for 10 min to allow for thorough separation of layers. The hexane layer was discarded and the aqueous layer filtered through nylon syringe filters (13 mm × 0.22 μm; Jaytee Biosciences Ltd., Herne Bay, UK) directly into amber salinized 2 mL HPLC. 

A reversed-phase Agilent 1200 series HPLC system with fluorescence detection was used to confirm the identity and quantify AFB_1_. This consisted of an in-line degasser, auto sampler, binary pump and a fluorescence detector (excitation and emission wavelength of 360 and 440 nm, respectively). Separation was achieved through the use of a C_18_ column (Agilent Zorbax Eclipse plus C_18_ 4.6 mm × 150 mm, 3.5 μm particle size; Agilent, Berks, UK) preceded by a guard cartridge with the same packing material. Isocratic elution, with a mobile phase that included methanol:water:acetonitrile (30:60:10, *v*:*v*:*v*), was performed at a flow rate of 1.0 mL/min. The injection volume was 20 μL. A set of standards was injected (1 to 5 ng AFB_1_, B_2_, G_1_ and G_2_ per injection) and standard curves were generated by plotting the area underneath the peaks against the amounts of AFB_1_ standard injected. Linear regression was performed in order to establish a correlation relationship (correlation coefficient, *R*^2^ = 0.99). 

### 4.4. Total RNA Extraction

For RNA-seq, tissue harvest was performed after 10 days using three replicates. This time frame was chosen because previous studies with both *A. flavus* and *A. parasiticus* suggested that gene expression of many of the biosynthetic genes was optimal after 8–10 days growth, although there does appear to be a sequential expression of groups of AF biosynthetic genes [39,40,41]. Studies on stored maize grain have also shown optimum AFB_1_ production at between 5–10 days at 0.98 a_w_ and 10 days at 0.95 a_w_ [42]. We have compromised and used 10 days in this study so that we can obtain molecular information and relevant toxin data. After 10 days of kernel infection (see above), 1 g of frozen milled maize was ground to powder using a mortar and pestle in the presence of liquid nitrogen, and then placed into a 2 mL extraction tube for isolation of total RNA. Total RNA was extracted using the RNAeasy Plant Mini kit (Qiagen, Hilden, Germany). One hundred milligram of the resulting powder was used for isolation of total RNA. The powder was resuspended in 1 mL lysis buffer supplemented with 10 μL β-mercaptoethanol in a 2 mL RNase free micro reaction tube. After vortexing, the tube was quickly frozen in liquid nitrogen. The sample was then thawed on ice. All further procedures were essentially the same as recommended by the manufacturer’s protocol.

### 4.5. RNA Sequencing

Library preparation for RNA sequencing was conducted using the NEB Ultra Directional RNA Library Prep Kit. Sequencing was performed on an Illumina HiSeq 2000 instrument. All samples had three biological replicates except the following samples: 30 °C/0.99 a_w_/350 ppm, 30 °C /0.99 a_w_/650 ppm and 37 °C/0.99 a_w_/650 ppm, which had two replicates, and 30 °C/0.99 a_w_/1000 ppm, 37 °C/0.99 a_w_/1000 ppm and 37 °C/0.99 a_w_/350 ppm which consisted of one replicate. Untrimmed sequencing reads were mapped to the *A. flavus* NRRL3357 (assembly JCVI-afl1-v2.0, http://www.ncbi.nlm.nih.gov/genome/360?genome_assembly_id=28730) reference sequence using GSNAP [43,44]. Reads (www.ncbi.nih.gov, accession ID: PRJNA380582) mapping to exons were counted using featureCounts [45] followed by differential expression testing with DESeq2 [46]. Genes were considered differentially expressed if they had an adjusted *p*-value < 0.05. Gene ontology and KEGG term enrichment was done using the GOSeq R Bioconductor package. The gene co-expression network was made using WGCNA (Weighted Gene Network Co-expression Analysis) with a signed network, the bi-weight mid-correlation method, and a soft-thresholding power of 10. Variance stabilized counts from DESeq2 were used as input to WGNCA. Genes that had an average read count of less than 10 across all samples were removed prior to WGNCA analysis. Principle Component Analysis plots and heat maps were generated using R. Venn Diagrams were created by BioVenn [47]. Gene ontology term enrichment analysis for WGCNA clusters was conducted using Fungifun2 [48]. Rendering of Gene Networks was performed using Cytoscape v3.4.0 [49]. KEGG annotation was produced through the BlastKOALA [50] service.

### 4.6. RNA Isolation, cDNA Synthesis and Quantitative PCR

RNA for qPCR was isolated using the RNeasy Plant Mini Kit (Qiagen) from infected maize kernels (see above). cDNA was synthesized according to the Omniscript RT kit protocol (Qiagen) using 500 ng of total RNA. The Bio-Rad CFX96 Real Time PCR Detection System (Bio-Rad, Watford, UK) was used for qPCR with TaqMan probes targeting two AF cluster genes, *aflD* and *aflR*, and the housekeeping gene β-tubulin gene [20,38]. Primer and probe sequences were obtained from Medina et al. [17]. Reactions from three biological replicates were prepared in 12.5 μL reaction mixtures in MicroAmp optical 96-well reaction plates and sealed with optical adhesive covers (Bio-Rad). The optimal thermal cycling conditions included an initial step of 10 min at 95 °C and all 45 cycles at 95 °C for 15 s, 55 °C for 20 s and 72 °C for 30 s. Quantification cycle (Cq) determinations were automatically performed by the instrument using default parameters. The expression ratio was calculated using the 2^−ΔΔ*C*t^ method [51]. 

## Figures and Tables

**Table 1:** Number of reads mapping to exons for all conditions tested.

		30 °C	37 °C
0.91 a_w_	350 ppm	3.37 × 10^7^	2.13 × 10^7^
	650 ppm	2.64 × 10^7^	3.00 × 10^6^
	1000 ppm	3.04 × 10^7^	6.84 × 10^6^
0.99 a_w_	350 ppm	1.04 × 10^7^	8.01 × 10^5^
	650 ppm	4.69 × 10^6^	2.24 × 10^6^
	1000 ppm	6.22 × 10^5^	1.03 × 10^6^

**Table 2:** KEGG Categories with a statistically significant overrepresentation of genes that are differentially expressed and their associated *p* values.

**Carbon Dioxide**		**Temperature**	
**(30 °C/0.99 a_w_/350 ppm vs. 30 °C/0.99 a_w_/1000 ppm)**		**(37 °C/0.99 a_w_/350 ppm vs. 30 °C/0.99 a_w_/350 ppm)**	
**KEGG Category**	***p*-Value**	**KEGG Category**	***p*-Value**
Glycolysis/Gluconeogenesis	0.003	Glycolysis/Gluconeogenesis	0.000
Purine metabolism	0.008	Starch and sucrose metabolism	0.002
Cysteine and methionine metabolism	0.009	Methane metabolism	0.017
Fructose and mannose metabolism	0.011	Riboflavin metabolism	0.024
Pyrimidine metabolism	0.025	Glutathione metabolism	0.024
Folate biosynthesis	0.030	Glycosphingolipid biosynthesis - globo series	0.025
Carbon fixation in photosynthetic organisms	0.041	Fructose and mannose metabolism	0.029
Inositol phosphate metabolism	0.049	Pentose phosphate pathway	0.043
**Water Activity**		**Combined**	
**(30 °C/0.91 a_w_/350 ppm vs. 30 °C/0.99 a_w_/350 ppm)**		**(37 °C/0.91 a_w_/1000 ppm vs. 30 °C/0.99 a_w_/350 ppm)**	
**KEGG Category**	***p*-Value**	**KEGG Category**	***p*-Value**
Riboflavin metabolism	0.001	Glycolysis/Gluconeogenesis	0.000
Inositol phosphate metabolism	0.002	Methane metabolism	0.002
Glyoxylate and dicarboxylate metabolism	0.011	Riboflavin metabolism	0.005
Purine metabolism	0.012	Inositol phosphate metabolism	0.009
Methane metabolism	0.019	Taurine and hypotaurine metabolism	0.014
Starch and sucrose metabolism	0.020	Fructose and mannose metabolism	0.014
Glycolysis/Gluconeogenesis	0.023	Starch and sucrose metabolism	0.015
Cyanoamino acid metabolism	0.031	Cyanoamino acid metabolism	0.017
Fructose and mannose metabolism	0.039	Purine metabolism	0.019
		Pyrimidine metabolism	0.022
		Ether lipid metabolism	0.049

**Table 3:** Known or putative gene clusters in *Aspergillus flavus* identified by the gene for their primary backbone enzyme. The fold change values indicate upregulation (positive number) or downregulation (negative number) according to RNA sequencing results. Clusters in bold indicate the gene is affected by both carbon dioxide levels tested.

				Effect of Carbon Dioxide		Effect of Water		Effect of Temperature	
				At 30 °C/0.99 a_w_:		At 30 °C, 0.91 a_w_ vs. 0.99 a_w_:		At 0.99 a_w_, 37 °C vs. 30 °C:	
#	*A. flavus* 3357	Name	SM* Product	650 ppm/350 ppm	1000 ppm/350 ppm	350 ppm	1000 ppm	350 ppm	1000 ppm
5	AFLA_006170	polyketide synthetase (PksP)	naphthopyrone	-	3.37	5.01	1.96	1.86	-
10	AFLA_016140	scytalone dehydratase (Arp1) (conidial pigment biosynthesis)	conidial pigment 1,8-dihydroxynaphthalene-melanin	-	-	−3.54	−2.21	-	-
15	AFLA_045490	dimethylallyl tryptophan synthase, putative	aflatrem, ATM2	2.50	-	-	-	-	-
**19**	**AFLA_060680**	**dimethylallyl tryptophan synthase**	**Unknown**	**−3.38**	**−4.09**	**−4.17**	**-**	**-**	**2.68**
20	AFLA_062860	polyketide synthase (PkfA)	3-(2,4-dihydroxy-6-methylbenzyl)-*O* rsellinaldehyde	1.35	-	-	−1.87	2.42	-
21	AFLA_064240	nonribosomal peptide synthase (*wykN*)	WYK peptidase inhibitor	-	2.23	-	−2.14	1.31	−2.19
**23**	**AFLA_066840**	**hybrid NRPS/PKS enzyme**	**Leporins**	**1.59**	**2.45**	**-**	**−3.27**	**-**	**−3.40**
35	AFLA_101700	NRPS enzyme (*lnaA*)	piperazines	-	2.15	-	−1.94	2.66	-
36	AFLA_104210	PKS-like enzyme, putative	dihydrocurvularin	-	-	−3.04	-	−2.94	-
39	AFLA_108550	polyketide synthase	monodictylphenone	-	-	-	-	-	-
41	AFLA_114820	polyketide synthase (fluP) (pksL2)	6-MSAi	-	1.55	-	−2.17	-	-
44	AFLA_116890	polyketide synthase (PkiA)	6-hydroxy-7-methyl-3-nonylisoquinoline-5,8-dione	-	−2.52	−3.49	-	-	-
54	AFLA_139410	polyketide synthase (*aflC*/*pksA*/*pksL1*)	aflatoxin	-	1.20	0.95	-	-	−2.05
55	AFLA_139490	hybrid PKS/NRPS enzyme	cyclopiazonic acid	-	-	−2.75	−5.39	-	−3.09
	**AFLA_125760**	**Class 2 Terpene Cyclase**	**Unknown**	**1.99**	**4.65**	**3.98**	**-**	**3.48**	**-**

* Putative product based on identity to characterized enzymes.

## Supplementary Materials

The following are available online at www.mdpi.com/2072-6651/10/1/5/s1, Table S1: SMURF. SMURF-designated secondary metabolic gene clusters in *A. flavus* and their associated log_2_ expression values, Table S2: Network Analysis. Genes identified as co-expressed by gene cluster network analysis and their corresponding differential expression values.

## References

[1] Vardon P., McLaughlin C., Nardinelli C. (2003). Potential economic costs of mycotoxins in the United States. Council for Agricultural Science and Technology (Cast). Mycotoxins: Risks in Plant, Animal, and Human Systems.

[2] Wu F. (2006). Mycotoxin reduction in Bt corn: Potential economic, health, and regulatory impacts. Transgenic Res..

[3] Wu F., Groopman J.D., Pestka J.J. (2014). Public health impacts of foodborne mycotoxins. Annu. Rev. Food Sci. Technol..

[4] Rigo K., Varga J., Toth B., Teren J., Mesterhazy A., Kozakiewicz Z. (2002). Evolutionary relationships within *Aspergillus section flavi* based on sequences of the intergenic transcribed spacer regions and the 5.8s rRNA gene. J. Gener. Appl. Microbiol..

[5] Kumeda Y., Asao T., Takahashi H., Ichinoe M. (2003). High prevalence of B and G aflatoxin-producing fungi in sugarcane field soil in Japan: Heteroduplex panel analysis identifies a new genotype within *Aspergillus* section flavi and *Aspergillus nomius*. FEMS Microbiol. Ecol..

[6] Arias R.S., Dang P.M., Sobolev V.S. (2015). RNAi-mediated control of aflatoxins in peanut: Method to analyze mycotoxin production and transgene expression in the peanut/*Aspergillus* pathosystem. J. Vis. Exp. JoVE.

[7] Cary J.W., Rajasekaran K., Brown R.L., Luo M., Chen Z.-Y., Bhatnagar D. (2011). Developing resistance to aflatoxin in maize and cottonseed. Toxins.

[8] Brown R.L., Menkir A., Chen Z.Y., Bhatnagar D., Yu J., Yao H., Cleveland T.E. (2013). Breeding aflatoxin-resistant maize lines using recent advances in technologies—A review. Food Addit. Contam. Part A Chem. Anal. Control Expo. Risk Assess..

[9] Ehrlich K.C. (2014). Non-aflatoxigenic *Aspergillus flavus* to prevent aflatoxin contamination in crops: Advantages and limitations. Front. Microbiol..

[10] Sanchis V., Magan N., Magan N., Olsen M. (2004). Environmental conditions affecting mycotoxins. Mycotoxins in Food.

[11] Abdel-Hadi A., Schmidt-Heydt M., Parra R., Geisen R., Magan N. (2012). A systems approach to model the relationship between aflatoxin gene cluster expression, environmental factors, growth and toxin production by *Aspergillus flavus*. J. R. Soc. Lond. Interface.

[12] Payne G.A., Thompson D.L., Lillehoj E.B., Zuber M.S., Adkins C.R. (1988). Effect of temperature on the preharvest infection of maize kernels by *Aspergillus flavus*. Phytopathology.

[13] Medina A., Schmidt-Heydt M., Rodriguez A., Parra R., Geisen R., Magan N. (2015). Impacts of environmental stress on growth, secondary metabolite biosynthetic gene clusters and metabolite production of xerotolerant/xerophilic fungi. Curr. Genet..

[14] Gilbert M., Mack B., Payne G., Bhatnagar D. (2016). Use of functional genomics to assess the climate change impact on *Aspergillus flavus* and aflatoxin production. World Mycotoxin J..

[15] Mitchell N.J., Bowers E., Hurburgh C., Wu F. (2016). Potential economic losses to the US corn industry from aflatoxin contamination. Food Addit. Contam. Part A Chem. Anal. Control Expo. Risk Assess..

[16] Misihairabgwi J.M., Ezekiel C.N., Sulyok M., Shephard G.S., Krska R. (2017). Mycotoxin contamination of foods in southern Africa: A 10-year review (2007–2016). Crit. Rev. Food Sci. Nutr..

[17] Medina A., Gilbert M.K., Mack B.M., GR O.B., Rodriguez A., Bhatnagar D., Payne G., Magan N. (2017). Interactions between water activity and temperature on the *Aspergillus flavus* transcriptome and aflatoxin B1 production. Int. J. Food Microbiol..

[18] Georgianna D.R., Fedorova N.D., Burroughs J.L., Dolezal A.L., Bok J.W., Horowitz-Brown S., Woloshuk C.P., Yu J., Keller N.P., Payne G.A. (2010). Beyond aflatoxin: Four distinct expression patterns and functional roles associated with *Aspergillus flavus* secondary metabolism gene clusters. Mol. Plant Pathol..

[19] Medina A., Rodriguez A., Magan N. (2014). Effect of climate change on *Aspergillus flavus* and aflatoxin B1 production. Front. Microbiol..

[20] Medina Á., Rodríguez A., Sultan Y., Magan N. (2015). Climate change factors and *Aspergillus flavus*: Effects on gene expression, growth and aflatoxin production. World Mycotoxin J..

[21] Hall R.A., De Sordi L., Maccallum D.M., Topal H., Eaton R., Bloor J.W., Robinson G.K., Levin L.R., Buck J., Wang Y. (2010). CO_2_ acts as a signalling molecule in populations of the fungal pathogen *Candida albicans*. PLoS Pathog..

[22] Romer D., Bollazzi M., Roces F. (2017). Carbon dioxide sensing in an obligate insect-fungus symbiosis: CO_2_ preferences of leaf-cutting ants to rear their mutualistic fungus. PLoS ONE.

[23] Koizumi H., Nakadai T., Usami Y., Satoh M., Shiyomi M., Oikawa T. (1991). Effect of carbon dioxide concentration on microbial respiration in soil. Ecol. Res..

[24] Gunterus A., Roze L.V., Beaudry R., Linz J.E. (2007). Ethylene inhibits aflatoxin biosynthesis in *Aspergillus parasiticus* grown on peanuts. Food Microbiol..

[25] Chang P.K., Ehrlich K.C., Fujii I. (2009). Cyclopiazonic acid biosynthesis of *Aspergillus flavus* and *Aspergillus oryzae*. Toxins (Basel).

[26] Gerhards N., Neubauer L., Tudzynski P., Li S.M. (2014). Biosynthetic pathways of ergot alkaloids. Toxins (Basel).

[27] Cary J.W., Uka V., Han Z., Buyst D., Harris-Coward P.Y., Ehrlich K.C., Wei Q., Bhatnagar D., Dowd P.F., Martens S.L. (2015). An *Aspergillus flavus* secondary metabolic gene cluster containing a hybrid PKS-NRPS is necessary for synthesis of the 2-pyridones, leporins. Fungal Genet. Biol..

[28] TePaske M.R., Gloer J.B., Wicklow D.T., Dowd P.F. (1991). Leporin A: An antiinsectan n-alkoxypyridone from the sclerotia of *Aspergillus leporis*. Tetrahedron Lett..

[29] Zhang C., Jin L., Mondie B., Mitchell S.S., Castelhano A.L., Cai W., Bergenhem N. (2003). Leporin B: A novel hexokinase II gene inducing agent from an unidentified fungus. Bioorg. Med. Chem. Lett..

[30] Alcazar-Fuoli L., Mellado E. (2012). Ergosterol biosynthesis in *Aspergillus fumigatus*: Its relevance as an antifungal target and role in antifungal drug resistance. Front. Microbiol..

[31] Dupont S., Lemetais G., Ferreira T., Cayot P., Gervais P., Beney L. (2012). Ergosterol biosynthesis: A fungal pathway for life on land?. Evolution.

[32] Payne G.A., Nierman W.C., Wortman J.R., Pritchard B.L., Brown D., Dean R.A., Bhatnagar D., Cleveland T.E., Machida M., Yu J. (2006). Whole genome comparison of *Aspergillus flavus* and *A. oryzae*. Med. Mycol..

[33] Nierman W.C., Yu J., Fedorova-Abrams N.D., Losada L., Cleveland T.E., Bhatnagar D., Bennett J.W., Dean R., Payne G.A. (2015). Genome sequence of *Aspergillus flavus* NRRL 3357, A strain that causes aflatoxin contamination of food and feed. Genome Announc..

[34] Ehrlich K.C., Mack B.M. (2014). Comparison of expression of secondary metabolite biosynthesis cluster genes in *Aspergillus flavus*, *A. parasiticus*, and *A. oryzae*. Toxins.

[35] Kim J.H., Yu J., Mahoney N., Chan K.L., Molyneux R.J., Varga J., Bhatnagar D., Cleveland T.E., Nierman W.C., Campbell B.C. (2008). Elucidation of the functional genomics of antioxidant-based inhibition of aflatoxin biosynthesis. Int. J. Food Microbiol..

[36] Linz J.E., Wee J., Roze L.V. (2014). *Aspergillus parasiticus* SU-1 genome sequence, predicted chromosome structure, and comparative gene expression under aflatoxin-inducing conditions: Evidence that differential expression contributes to species phenotype. Eukaryot. Cell.

[37] Donner M., Atehnkeng J., Sikora R.A., Bandyopadhyay R., Cotty P.J. (2010). Molecular characterization of atoxigenic strains for biological control of aflatoxins in Nigeria. Food Addit. Contam. Part A Chem. Anal. Control Expo. Risk Assess..

[38] Abdel-Hadi A., Carter D., Magan N. (2010). Temporal monitoring of the *nor-1* (*aflD*) gene of *Aspergillus flavus* in relation to aflatoxin B(1) production during storage of peanuts under different water activity levels. J. Appl. Microbiol..

[39] Schmidt-Heydt M., Abdel-Hadi A., Magan N., Geisen R. (2009). Complex regulation of the aflatoxin biosynthesis gene cluster of *Aspergillus flavus* in relation to various combinations of water activity and temperature. Int. J. Food Microbiol..

[40] Schmidt-Heydt M., Rufer C.E., Abdel-Hadi A., Magan N., Geisen R. (2010). The production of aflatoxin B1 or G1 by *Aspergillus parasiticus* at various combinations of temperature and water activity is related to the ratio of *aflS* to *aflR* expression. Mycotoxin Res..

[41] Schmidt-Heydt M., Magan N., Geisen R. (2008). Stress induction of mycotoxin biosynthesis genes by abiotic factors. FEMS Microbiol. Lett..

[42] Mohale S., Medina A., Rodriguez A., Sulyok M., Magan N. (2013). Mycotoxigenic fungi and mycotoxins associated with stored maize from different regions of Lesotho. Mycotoxin Res..

[43] Wu T.D., Nacu S. (2010). Fast and SNP-tolerant detection of complex variants and splicing in short reads. Bioinformatics.

[44] Wu T.D., Watanabe C.K. (2005). GMAP: A genomic mapping and alignment program for mRNA and EST sequences. Bioinformatics.

[45] Liao Y., Smyth G.K., Shi W. (2014). featureCounts: An efficient general purpose program for assigning sequence reads to genomic features. Bioinformatics.

[46] Love M.I., Huber W., Anders S. (2014). Moderated estimation of fold change and dispersion for RNA-seq data with DESeq2. Genome Biol..

[47] Hulsen T., de Vlieg J., Alkema W. (2008). BioVenn—A web application for the comparison and visualization of biological lists using area-proportional Venn diagrams. BMC Genom..

[48] Priebe S., Kreisel C., Horn F., Guthke R., Linde J. (2015). FungiFun2: A comprehensive online resource for systematic analysis of gene lists from fungal species. Bioinformatics.

[49] Shannon P., Markiel A., Ozier O., Baliga N.S., Wang J.T., Ramage D., Amin N., Schwikowski B., Ideker T. (2003). Cytoscape: A software environment for integrated models of biomolecular interaction networks. Genome Res..

[50] Kanehisa M., Sato Y., Morishima K. (2016). Blastkoala and ghostkoala: KEGG tools for functional characterization of genome and metagenome sequences. J. Mol. Biol..

[51] Livak K.J., Schmittgen T.D. (2001). Analysis of relative gene expression data using real-time quantitative PCR and the 2(-delta delta C(t)) method. Methods.

